# Correction to: Genetic Engineering of *Escherichia coli* W for Linalool Production Using Beet Juice as the Sole Carbon Source

**DOI:** 10.1007/s12010-025-05480-1

**Published:** 2025-11-18

**Authors:** Matthew McKillop, Taufiq Nawaz, Liping Gu, Shah Fahad, Ewumbua Monono, Ruanbao Zhou

**Affiliations:** 1 Biology and Microbiology, South Dakota State University, Brookings, SD 57007 USA; 2 Agricultural and Biosystems Engineering, North Dakota State University, Fargo, ND 58102 USA

**Correction to: Applied Biochemistry and Biotechnology**

10.1007/s12010-025-05439-2

The original online version of this article was revised. The authors found two (2) errors for this article enumerated below.

1. Article title.
  Original: Genetic Engineering of *Escherichia* *Coli* W for Linalool Production Using Beet Juice as the Sole Carbon Source
  Corrected: Genetic Engineering of *Escherichia* *coli* W for Linalool Production Using Beet Juice as the Sole Carbon Source
2. Dr. Shah Fahad was incorrectly listed as a corresponding author. The correct corresponding authors are:
  Dr. Liping Gu
  Prof. Dr. Ruanbao Zhou

This correction aligns with the author information provided in both the original submission and the revised submission.

